# The Culture of Violent Talk: An Interpretive Approach

**DOI:** 10.3390/socsci9070120

**Published:** 2020

**Authors:** Pete Simi, Steven Windisch

**Affiliations:** 1 Department of Sociology, Chapman University, One University Drive, Orange, CA 92866, USA; 2 Department of Criminal Justice, Temple University, Philadelphia, PA 19122, USA

**Keywords:** white supremacy, threat assessment, identity, terrorism, culture, violence

## Abstract

One of the defining characteristics of extremist movements is the adherence to an ideology highly antagonistic to the status quo and one that permits or explicitly promotes the use of violence to achieve stated goals and to address grievances. For members of extremist groups, talk is one of the most concrete manifestations of how adherents communicate their ideas to each other and the general public. These discussions, however, do not necessarily involve a direct correspondence between words and future behavior. To better understand the culture of violent talk, we investigate how white supremacist extremists use these discussions as a rhetorical device that provides them with a sense of doing and an opportunity to express their frustrations and anger. Our analysis is grounded primarily in the ethnographic data we collected on a variety of US white supremacists since 1997. Our investigation offers important insight regarding the interactional qualities of extremist culture as well as policy implications regarding the assessment process.

## 1. Introduction

Talk is one of the most concrete manifestations of how adherents of an extremist movement communicate their culture to each other and the general public.^1^ The words extremists speak and write is often understood as a proxy for behavior. The content of violent talk, however, can be highly misleading, especially if there is an assumption that a straightforward one-to-one relationship exists between words and action or even between words and meaning. In some cases, talk involves “figures of speech” (Drew and Holt 1998), where extremists rely on dramatic phrases to express emotions or a general frame of mind. The figurative dimension of talk highlights the degree to which words are symbolic and stand in contrast to viewing talk as a transparent indicator of future behavior. However, there has been little effort to use symbolic interactionism to examine the potential disjuncture between talk and action among extremists (for exception see Mitchell 2003). Moreover, although a large literature examines the structure and content of fascist discourse (Billig 1978; Falasca-Zamponi 1997; Wodak and Richardson 2013), informal conversations among extremists have received only episodic attention especially since most studies of extremism does not include this type of data. Considering this, we ask the following: what is the relationship between violent talk and extremist culture? More specifically, we focus on the contemporary US white supremacist movement (WSM) to explore how members express violent talk across different spatial locations.

The goal of this study is to emphasize the importance of “violent talk” as a form of action that provides actors with a sense of doing (Berger and Luckmann 1966) and an opportunity to express the frustrations and anger that typically characterize an extremist identity. By violent talk, we refer to utterances by extremists who express an ideology and invoke the use of violence as part of those expressions. Violent talk can occur during informal face-to-face discussions as well as through virtual conversations on platforms like *Facebook* and, more recently, *Telegram* and *Discord*. We view violent talk as part of a performance that provides actors with an opportunity to achieve consistency between ideas and behavior (Blumer 1969; Goffman 1959). In this sense. the effects of violent talk are indeterminate. In one sense, violent talk can reinforce the value of violence and its importance as a cultural and political practice. From this perspective, violent talk is central to the subculture of violence which increases the likelihood of its use. In another sense, however, violent talk can serve as a “stand in” or substitute for violent behavior. The indeterminacy of violent talk is one of the most important findings of this study.

Our analysis is grounded primarily in the ethnographic data we collected on a variety of white supremacist activists since 1997. Theoretically. the analysis draws from a tradition of symbolic interactionist research focused on the interactive qualities of talk (Garfinkel 1967). In particular, we employ theoretical and empirical insights from interactionist research that distinguishes between “sentiments and acts” (Deutscher et al. 1993; Festinger 1964) as well as “identity talk” (Hunt and Benford 1994; Lichterman 1999; Snow and Anderson 1987), which is devoted to understanding how actors use talk to communicate a particular worldview and manage different aspects of their identity. Identity talk is especially crucial for actors managing a stigmatized identity. In the next sections, we provide an overview of contemporary right-wing extremism with specific focus on white supremacist extremism in the US, followed by an examination of the relationship between talk and violence.

## 2. Literature Review

### 2.1. Contemporary Right-Wing Extremism in the US

Right-wing extremism (RWE) is highly contested among academics and the public (Kaplan and Bjørgo 1998; Von Mering and McCarty 2013; Merkl and Weinberg 2003; Rydgren 2004). We describe RWE as representing a constellation of various overlapping and distinct movements whose ideological orientations cover a wide range of views regarding racial/ethnic supremacy, anti-government opposition, anti-Semitism, xenophobia, homophobia, and misogyny (Berlet and Lyons 2000; Diamond 1995; Godin et al. 2013; Smith 1994; Weinberg 1998).

The election of Barack Obama, America’s first African American President, along with several other “hot button” issues, has led to a rapid increase in RWE during the past decade (Beirich and Potok 2009). Following Obama’s election. the emergence of the Tea Party Movement along with a host of other ancillary movements such as the “Birthers” reflect a growing polarization now fully apparent in mainstream electoral politics and, in particular, among segments of the Republican Party (Linkins 2012). The 2016 election of Donald J. Trump is likely the most telling example of this polarization. Trump’s candidacy and administration have explicitly incorporated white supremacist rhetoric and memes. The polarization, however, extends well beyond elected officials and leading pundits as some 20% of individuals who identify as “Republicans” viewed President Obama as the “Antichrist” (Bennett-Smith 2013).

Engrained in the current landscape of polarized politics is the contemporary US WSM, which represents one of the most radical segments of RWE. The WSM represents a broad constellation of several overlapping branches, including the Ku Klux Klan, Christian Identity, national socialists, and racist skinheads (Simi and Futrell 2015). Although differences exist, there is broad agreement on various fundamental doctrines (Burris et al. 2000). Foremost is the belief that Whites are part of an innately superior biogenetic race (i.e., “master race”) that is under attack from homosexuality, “race-mixing,” and multiculturalism (Berbrier 2000). White supremacists desire a racially exclusive world where non-Whites and other “sub-humans” are segregated, or at least subordinated to “Aryan authority” (Ferber 1998). White supremacists idealize conservative, traditional male-dominant heterosexual families and loathe inter-racial sex, marriage, and procreation (Simi and Futrell 2015). White supremacists rally around the “14 Words” slogan (i.e., “*We must secure the existence of our people and a future for white children.*”) and more recent memes such as “Multiculturalism is Codeword for Anti-White” and “It’s Okay to be White.” While these elements remain an essential fixture of the WSM. the past decade has been characterized by growing diversity of groups, new leaders, and the development of new organizations. For example. the emergence of the “alt-right” signaled a general effort among white supremacists to rebrand their ideology in a way that is more palpable to mainstream White America (Futrell and Simi 2017). Moreover. the use of cyberspace, including various social media platforms, has been especially helpful in the “cross-fertilization” of ideological and organizational precepts.

The culture of contemporary WSM is highly emotive and relies significantly on symbolic iconography. While the emotional landscape of this culture ranges from anger, resentment, and fear to pride, passion, and love, there is little question that negative emotional states are especially prominent (Futrell et al. 2006; Virchow 2007). Violence is at the core of the WSM as ideological precepts encourage violence as a form of self-defense necessary to prevent the eradication of the white race (Ferber 1999). As such, a substantial dimension of a white supremacist identity involves promoting violence, but like most violent movements, catalyzing violent action is much more difficult than expressing violent words (Windisch et al. 2018; Windisch et al. forthcoming). The discrepancy between belief and action means that interpreting the role of violent talk is paramount, while at the same time, highly complex.

### 2.2. Interpreting Violent Talk

Talk is a routinized element of social interaction and is indexical in that each word stands for something. Taken together, a string of words exchanged between two or more persons becomes a conversation. Talk and action are central components of human behavior, and social scientists have focused extensively on whether words determine action. During a conversation, individuals produce statements, and in some cases, these statements reflect aspects of a person’s identity (Mead 1934). These statements, however, do not always translate into action. Although there are instances where a strong relationship exists between sentiments and acts, a fundamental disjuncture often exists between “what people say” and “what people do” (Deutscher et al. 1993; Pestello and Pestello 2000). The lack of consistency between sentiments and acts may arise for a variety of reasons.

One issue involves the micro-structure of conversation and the institutional nature of talk. Emerging in the 1970s, conversation analysis (CA) became the premier method for understanding the structure and interactive qualities of talk (see Garfinkel 1967; Sacks 1972, 1973, 1974). Using CA, researchers have identified various dimensions of talk, such as “turn-taking” and “filling voids” (Sacks et al. 1974; Schaffer 1983). For example, in a typical conversation, actors exchange speaking turns in a relatively orderly fashion to minimize “awkward silences” and prevent interruptions or speaking over each other (Sacks et al. 1974). Silent pauses are awkward because the chatter of talk is ingrained (at least among Americans) (Jaworski 1997). In situations where a conversation stalls and silence develop, actors may end the interaction, continue by discussing prior matters, or start a new conversation (Hoey 2018). From this perspective. the function of talk is sometimes little more than filler. In other words. the content of a conversation is not necessarily determined by behavioral intentions, but rather, by conversational expectations (Schegloff 2000). Words within a conversation may be little more than scaffolding to buttress the completion of a specific exchange with little or no intention of furthering a course of behavioral action. As such. the recognition that expressing white supremacist ideology and invoking the use of violence can be used to merely hold a conversation together should give pause to those who assume a consistent relationship between words and action among extremists.

Another issue pertains to how actors use talk to communicate and manage different aspects of their collective identity (Blumer 1969). Identity among social movement activists defines in-group/out-group relationships, organizes formal ceremonies or rituals, maintains morale, and articulates shared ideologies (Klapp 1969; Turner and Killian 1957). Since alignment between personal and collective identities is not always straightforward and identities are products of social interaction, actors use “identity talk” to construct, interpret, and communicate their identities via verbal utterances, gestures, acts, dress, and appearances (Hunt and Benford 1994; Lichterman 1999; Snow and Anderson 1987). Identity talk is used as a discursive practice to demonstrate that an individual’s identity is consistent with the perceived collective identity of the movement (Hunt and Benford 1994). Identity talk is viewed as rhetoric that is constructed in accordance with group-specific guidelines, varies temporally, and is redefined continuously as new experiences unfold (Snow and Machalek 1984). From this perspective, conversations among white supremacists can be viewed, in part, as a performance that provides an opportunity to achieve consistency between their personal and collective identity (Goffman 1959). In this way. the exchange of violent talk is an action itself that white supremacists use to symbolize their collective identity and communicate their identification with a particular worldview.

What is surprising is that people assume extremists are unlikely to exhibit inconsistencies between what they say and do because they are “true believers” (Hoffer 1951). This rather inflexible view of extremism is contradicted by a number of scholars who emphasize the distinction between cognitive and behavioral radicalization such that most individuals who adopt extremist beliefs and either explicitly or implicitly support violence do not actually commit violence themselves (e.g., see Borum 2011; McCauley and Moskalenko 2011; Khalil et al. 2019). In addition, ethnographic evidence also suggests extremists are highly adaptive and find creative strategies for negotiating inconsistencies (Simi and Futrell 2009). Moreover, extremists may have little, if any, awareness of an inconsistency, and thus, may not see the need to reconcile what appears from the outside as a contradiction (Pestello and Pestello 2000). Acknowledging the performative nature of extremism is not meant to diminish the extent or severity of violence committed by extremists. Rather, a performative perspective emphasizes nuance and is skeptical of assuming a direct correspondence between what people say and what people do. In some cases, violent talk may be a necessary condition for extremist violence, but many who engage in violent talk will not transition to actual violence (Simi and Windisch 2018; Windisch et al. forthcoming).

Finally. the local character of conversation means that apart from certain structural features of talk, conversations are contextual and operate according to the logic of “this doesn’t go beyond us.” In this sense. the content of conversation is unique such that any understanding of the words expressed is entirely contingent on a variety of contextual factors. The form by which conversations occur may have institutionalized expectations that cut across situations, but how these expectations are met varies widely. Although conversations can resemble hyperlinks on the Internet. the local nature of talk creates difficulties navigating from one conversation to the next. A slight cue present in one conversation, but missed in another, can radically alter how these same words are understood. In short, conversations may not translate clearly from one social-spatial location to another. As such. the socio-spatial location of these conversations provides a significant opportunity to consider the situational nature of violent talk. We focus on several of these spaces in the remaining sections.

## 3. Methods

Our analysis is grounded primarily in the ethnographic data we collected on a variety of white supremacist activists and groups between 1997 and present. We used a multi-method approach (Snow and Anderson 1993), which included participant observation in a variety of settings and one to three hour in-depth face-to-face and telephone interviews with 56 WSM activists. Of the 56 interviewees, 40 were with male activists, and 16 were with female activists. The activists’ ages ranged from 15–25 years (n = 7), 26–35 years (n = 26), 36–45 years (n = 8), 46–55 years (n = 9), and 55 and over (n = 6). Of those, 14 were movement leaders, and 42 were rank-and-file activists. We conducted 39 follow-up interviews with the primary movement contacts, for a total of 95 interviews. We also performed a content analysis of WSM texts such as newsletters, websites, Internet discussion groups, and radio broadcasts.

We conducted ethnographic fieldwork with Christian Identity members in Utah and Arizona, Aryan Nations members in Idaho, and a variety of white supremacists in Southern California, including significant leaders such as Tom Metzger (founder of White Aryan Resistance). The range of events observed in Utah and Arizona included 23 house visits lasting from one to three days and various social gatherings such as parties, music concerts, court hearings, and leisure activities (e.g., hikes). We also conducted fieldwork at Aryan Nations’ former headquarters on four different occasions for three to five days in length, which provided opportunities to observe three Aryan Nations World Congresses and more informal gatherings (e.g., a wedding, prayer services, press conferences). Finally, fieldwork in Southern California included observations of various social gatherings and 22 visits in white supremacists’ homes ranging from two days to five weeks. Participant observation in these settings allowed for, among other things, an examination into the structure and content of violent talk exchanged during informal conversations among extremists and the role violent talk plays in terms of constructing and sustaining collective identity.

We selected interview subjects through snowball and purposive sampling strategies, which enabled us to access a wide range of activists, networks, and groups within the WSM. The sample included members of networks active in 18 states.^2^ Specific organizations represented include White Aryan Resistance, Aryan Nations (AN), and local branches of AN, Hammerskins, National Alliance, Ku Klux Klan, and various smaller white supremacist groups.^3^ Our interviews focused on the types of activism individuals had engaged in. the movement strategies advocated within and across the groups, and the meanings activists attached to various types of movement participation. Participant observation and interviewing allowed for the close examination of a wide range of political activism, which is not available through sole reliance on secondary sources and movement propaganda (Blee 1996). We also analyzed secondary sources for evidence that would either corroborate or contradict the insights about the movement we gleaned through primary interviews and observational data. Our multi-method approach allowed for triangulation across an array of data (Denzin 1978).

We analyzed the ethnographic data gathered through our content analysis using a modified grounded theory approach (Charmaz 2006; see also Berg 2007), which allows researchers to combine a more open-ended, inductive approach while also relying on existing literature and frameworks to guide the research. The initial data coding began by reading entire interview transcripts line-by-line to determine differences and similarities within and across the sample. Inductive codes emerged from the initial phase of line by line analysis (Lofland et al. 2006). Deductive codes were extracted from scholarly literature on talk, identity, violence, and related topics. After the initial codes were developed, we compared and contrasted themes, noting relations between first-level data and more general categories (Glaser and Strauss 1967; Miles and Huberman 1994).

Several limitations of this study are important to mention. First, we encountered methodological difficulties that we mainly attribute to WSM members’ preference for secrecy and, at times, for illegal activity. We relied on non-probability sampling due to the hidden character of the population (see Heckathorn 1997, 2002). Entry into the movement’s groups is difficult, and we obtained many of our interviewees through an introduction by our initial movement contacts. Also, as the WSM appears to be diversely structured with multiple centers and levels of activism, generalizations about the movement will remain modest and tentative.

## 4. Results

### 4.1. Socio-Spatial Location of Violent Talk

In a series of publications by Pete Simi and Robert Futrell. the authors focused on the role of “free spaces” (Futrell and Simi 2004; Simi and Futrell 2006; Simi and Futrell 2009; Simi and Futrell 2015) in sustaining collective identity and facilitating movement persistence (also see Couto 1993; Evans and Boyte 1992; Polletta 1999). Free spaces refer to small-scale settings that provide activists with autonomy from dominant groups where they can nurture oppositional movement identities. An essential component of these settings includes the different types of identity talk that occur, which helps participants align their personal identities with their collective identities. Identity alignment, in turn, helps movement persistence by increasing the likelihood individual activists will sustain involvement (Simi and Futrell 2015).

There are two broad types of free spaces: local and translocal. Local free spaces are characterized as small, interpersonal networks where members reaffirm and transmit commitment to the movement’s goals and discourage sympathy toward mainstream cultural codes, which helps create and sustain a collective identity. Many of these practices are relatively hidden as otherwise mundane activities performed in private settings such as family homes, Bible study groups, and crashpads (Futrell and Simi 2004). Other types of informal gatherings provide a space where members enact relationships they envision as ideal and reflective of broader movement ethos (e.g., exclusion of non-whites). Limited in reach and scope to relatively small geographic areas, local free spaces cannot alone provide the network connections activists require to maintain their culture beyond the bounds of small interpersonal networks.

In contrast, translocal free spaces refer to larger-scale occasions that draw otherwise unconnected local activists into broader webs of extremist culture and identity through physical and virtual socio-spatial locations. For example, intentional communities (e.g., Aryan Nations compound, Elohim City), music festivals, Aryan Nations Congresses, and other similar events are typical translocal free spaces. Other types of translocal spaces include settings in cyberspace such as chatrooms, forums, and social networking sites. Compared to the smaller, more insular nature of local free spaces, translocal free spaces involve larger gatherings across a wider geographic area and attract participants characterized by both weak and strong ties (Granovetter 1973). By participating in translocal free spaces, local activists feel they are not alone, but rather, part of a larger, ongoing movement culture that helps reinforce solidarity, faith, and commitment to the cause. Moreover, such alignment provides activists with a model of the world they seek to create through their activism. Together, local and translocal free spaces create a bi-level *infrastructure of space* that contributes to the persistence of activism by encouraging distinct kinds of network ties and activities through which members sustain collective identity. Maintaining free space infrastructure is critical to the persistence of the WSM. Without the contributions of local and translocal free spaces, white supremacist networks that sustain activism would atrophy.

Across both of these socio-spatial settings, participants consistently displayed violent talk. The violent talk that occurred in these spaces communicated ideological commitments, traced in-group and out-group boundaries, and defined grievances. Participants embedded violent talk into their narratives (Polletta 2006) as they exchanged and discussed “racial awakenings” (Blee 2002), “fortifying tales” (Polletta 1998), racist humor (Billig 2001), and solutions to social problems. The prescription of violent solutions typically involved either highly elaborate acts of genocide or brutal acts of interpersonal violence. Despite differences in the characteristics of the settings. the violent talk was markedly similar. For instance, violent talk at organized music festivals resembled spontaneous events such as a small informal gathering of white supremacists watching television. The fact that violent talk was pervasive among white supremacists tells us little, however, about the role of violent talk or how actors defined and interpreted violent talk. In the next section, we discuss each type of free space and provide examples of the talk that occurred in these milieus.

### 4.2. Violent Talk in Local Free Spaces

During fieldwork, violent talk represented a type of “ordinary conversation” (Heritage 1984) that occurred as part of social interaction. We observed individuals discussing violent talk in local free spaces such as homes and house parties. Within these settings, white supremacists felt “free” to express deviant ideas that challenged the status quo, including violent solutions.

#### 4.2.1. White Supremacist Homes

The home constitutes a space where parents trace in-group/out-group boundaries and transmit white supremacist sentiments. White supremacists describe the family as the “foundation” of the WSM in terms of its role in ensuring the persistence of collective identity across generations since it is the earliest means of racial socialization. With a few exceptions, parenting is perceived as a form of activism and an opportunity to help build the next generation (Simi et al. 2016). Parents envision helping their children develop a sense of commitment to movement ideals as the family becomes a haven where there is almost complete freedom to talk about these ideals. In turn, parents talk about their children becoming “Aryan warriors” who are going to help fight the race war. For example, during an informal get-together among Southwest Aryan Separatists (SWAS) members, a conversation emerged regarding the threat of government standoffs. During the conversation, a father of three stated:

All my kids, you know, we’re shooting it out, we’re staying. My boys, they say “Dad we’re going to hold our guns and shoot back and if you and Mom get killed, we’re gonna shoot it out.”

(James, SWAS, 30 March 1999)

Similarly, an impromptu conversation among two parents at a Southern California family barbeque involved the following:

Diane: “So what would you do if Jenny [his daughter] brought a nigger home [laughs while finishing sentence]?”

Rich: “You know there’s no way she’d ever do that. But if she did, I’d kick her out of the house after I slit the niggers’ throat.” (SoCal Aryans, 3 June 2004)

Both statements involve an expression of white supremacist identity and, as such, can be viewed as accounts (Scott and Lyman 1968; see also Orbuch 1997) provided by each actor to others (and themselves) that demonstrate consistency between beliefs and action. An account involves a “linguistic device employed whenever an action is subjected to evaluative inquiry” (Scott and Lyman 1968, p. 46). The above statements reflect situational responses to hypothetical scenarios where the actor anticipated potential judgment from other white supremacists based on his/her assessment of what a white supremacist should do in a given scenario. In each statement, parents express the idea that their children are committed to the cause. In the first example, James reports what his children have allegedly said as a means to demonstrate his family’s ideological commitment. In the latter example, Rich expresses the idea that his child would never pursue an interracial relationship. However, in the unlikely event that his child did such a thing, there would be lethal consequences. Each scenario provided the actors with an opportunity to enact a type of idealized rhetorical-based role performance. White supremacist parents deploy children like props in fantasy-driven scenarios that involve government standoffs and other aspects of the “racial holy war” (referred to as RAHOWA) such as “racial cleansing” to reinforce racial boundaries and punish those who violate these hierarchies, including their children.

It is easy to overlook the hypothetical nature of these scenarios and responses. These statements reflect parents’ perception that part of being a parent is raising their children in accordance with movement ideals. As such, these accounts are meant to demonstrate consistency between beliefs and actions. That consistency, however, may not extend beyond the bounds of these situated interactions. Acknowledging the performative nature of these interactions does not diminish the severity of these accounts, but rather, challenges the conventional wisdom that assumes a direct correspondence between what people say and what people do. Although the content of violent talk suggests an unproblematic relationship in terms of establishing consistency between sentiments and action. the radical and totalizing nature of extremism means that achieving consistency is anything but unproblematic. For example. the hypothetical presented to Rich came to fruition some eight years later as his daughter eventually married a “non-White” Latinx person and gave birth to the couple’s child. The daughter’s relationship, along with several other factors, encouraged Rich to disavow his white supremacist beliefs and affiliations. Today, Rich has a close relationship with his daughter, son-in-law, and grandchild. The point is not that Rich was unserious about using violence to express his ideology, but rather, his words proved to be a poor barometer of the actions he ultimately took when faced with that very same scenario. The relationship between Rich’s words and eventual behavior is complicated by the lengthy-time period between his statement and when the scenario came to fruition. It is certainly possible that had the scenario emerged in closer proximity to when he expressed these sentiments. the response to his daughter’s romantic relationship may have been quite different.

#### 4.2.2. House Parties and Informal Gatherings

White supremacists also use house parties to exchange violent talk. Within these settings, participants described what it means to be “Aryan,” the injustices the White race faces, how they became enlightened to the cause, and celebrations of small victories against the Zionist Occupational Government (referred to as ZOG) and other racial enemies. Violent talk amplifies solidarity and purpose among veterans and introduces newcomers to their culture. For example, at a luncheon with about twenty attendees in Northern Idaho, a Nazi biker visiting from the Midwest was asked by a younger member in his late teens or early twenties about the “racial situation” in the biker’s hometown. The biker replied with the following:

I think it’s time to start digging holes and fucking bulldoze all the niggers in just fucking get rid of ‘em. It’s so bad out there you wouldn’t believe it.

(Aryan Nations, 13 April 1997)

Fellow attendees greeted the biker’s comment with nods of approval and laughter, along with a series of other comments. For instance, a man in his early sixties responded by recalling his time living in Watts, California:

I lived in this motel surrounded by niggers literally everywhere. They would bang on my door all hours of the night. I couldn’t take it. I used to dream about just [starts making shooting sounds with his mouth], you know, clean the filth up.

(Aryan Nations, 13 April 1997)

Although crude and indicative of pathological thinking, each of these statements are also examples of figurative speech (Drew and Holt 1998). The violent talk in these spaces relates to the efficacy of violence but in a figurative sense. While highly disturbing, these examples are not necessarily indicative of concrete proposals to commit violence. Pointing out the figurative quality of these statements is not meant to trivialize the role of violent talk in the radicalization process or to diminish the offensive quality of these statements, but rather, serves as a reminder that talk, including offensive and disturbing talk, is often figurative rather than literal. Moreover. the relationship between free spaces and violence is paradoxical. On the one hand. the inspiration for white supremacist violence emanates from the culture of hate that endures in these free spaces. Yet. the existence of free spaces also provides social ties that may help constrain the most radical actions. In fact, we observed many individuals stressing the importance of staying true to the white supremacist vision while also discouraging overt acts of violence that draw negative attention. The risk of repression increases the likelihood of losing supportive social environments where white supremacists find positive status and experience feelings of joy, trust, reciprocity, racial kinship, love, and belonging with like-minded others. In this way, free spaces may act as pressure release valves that allow individuals to express frustrations among themselves through violent talk rather than in overt acts of violence against “racial enemies.”

### 4.3. Violent Talk in Translocal Free Spaces

In addition to local free spaces, violent talk in translocal free spaces contributes to the persistence of activism by encouraging distinct kinds of network ties and activities through which members sustain collective identity. Although the microstructural characteristics of these spaces differ, violent talk that occurs in translocal free spaces such as music festivals and online environments was generally the same kind of talk that occurred in local free spaces. In the next section, we provide examples of the violent talk that occurred in these translocal free spaces.

#### 4.3.1. Music Festivals and Formal Gatherings

Although translocal free spaces tend to draw from broader movement networks. the type of violent talk in these spaces was similar to the violent talk observed in local free spaces. More specifically, violent talk among white supremacists who were close friends resembled the violent talk that occurred among white supremacists who were strangers. Such a finding is less surprising since these spaces share similar structural characteristics: exclusive, intimate, and rooted in enduring relationships—all qualities that encourage members to express their radical racist ideologies safely and openly. For example, during a break at an Aryan Nations Congress, seven white supremacists who were previously unacquainted sat around the bunkhouse discussing different scenarios for enacting violence.

Will: “I’d like to find an island with shark infested waters surrounding it just, plunk, drop all the fags, all the queers. the Jews. the niggers. the spics and just leave ‘em there if they didn’t kill each other first the sharks would have fun if they tried to leave.”

Russ: “We need to start acting like Mongoose and start picking off the enemies one at a time, so they don’t know what’s hitting ‘em.” (Aryan Nations, 3 July 1999)

The conversation continued for another ten minutes focusing on similar ideas for violence until the break was over, and it was time to return to the Aryan Nations church to attend another round of speeches. Like the kinds of violent talk at house parties. the above interaction conveyed a fantasy-driven scenario (Billig 2001). Such discussions may refer to events that seem unlikely, yet their content is marked by racist violence performed by assumedly real racist actors. And, even more complicated, versions of the scenarios have been enacted by past violent white supremacists who are widely glorified as “martyrs” across the movement.

The above example underscores the central argument of this paper by illustrating the social function of violent talk. In general, talk provides social actors with a type of “gel” creating cohesion and is often linked to ritualistic performances (Heritage 1984). In the case of white supremacists, a central component of their talk involves violence which they use reinforce boundaries between themselves and nonmovement “others,” and recruit new members. While nonverbal forms of communication (e.g., dress, gestures) are essential in cultivating such relationships, violent talk is a consistent mechanism used to signify their commitment to the White race and demonstrate their resistance to perceived threats. For example, openly displaying Aryan-themed tattoos declares one’s faith to the White race (Simi and Futrell 2015), but telling stories explaining the significance of the tattoo as it relates to committing violence establishes the meaning it has for the person who feels they have “earned” the ink.

#### 4.3.2. Cyberspace

Cyberspace represents another type of translocal free space white supremacists used to connect with a larger number of like-minded adherents. Many websites are channels to real-world movement spaces, giving activists opportunities to connect with others (Simi and Futrell 2006). As part of cyberspace interaction, white supremacists sometimes concretize violent talk by posting models of real-world violence. Members post links to articles on hate crimes and ethnic conflicts around the world by framing them as inspirational stories to help “keep the faith.” For instance, a Free Your Mind Productions forum member posted a news article with photos and a video clip of a Gay Rights march in Belgrade, Serbia. The ensuing discussion focused on the melee that erupted during the march when neo-Nazis and Serbian nationalists attacked and severely injured several of the marchers. As a small sample of the discussion thread demonstrates, members enthusiastically hailed the violence:

“Fag bashing by moonlight oh god don’t it feel so right … Damn those pics are great. If anyone deserves a good beating, it’s that lot. Takes me back to the good ole days of my youth. Three cheers for the brothers taking care of business.”

(Battlefront, 13 February 2004)

“Fag bashing? I don’t know. I’d be afraid to get their blood on me. Really if you think about it, God only knows what these people got and all it takes is some blood getting splashed on your person.”

(NordicThunder, 13 February 2004)

“You’re supposed to bash fags with bricks. I feel the same way about nigger blood. I always carry leather gloves. Hate Crimes Pass the Time!”

(Odinsdaughter, 13 February 2004)

“Don’t waste good leather gloves on a nigger. What if you can’t get the stains out? Just use cheap fake leather mits and a long steel pipe or crowbar. That way you can whack them in the head from a distance and not even get any on you.”

(Aryanfront, 13 February 2004)

In this example, violent talk provides linkages between users of this digital forum and offers a cathartic opportunity to articulate hostility against “fags” and reminisce about the “good ole days.” Compared to the discussion at the Aryan Nations bunkhouse. the original reference point in these statements was an actual incidence of white supremacist violence. However, these responses are similar to the various hypothetical scenarios previously discussed in that there is a fantasy-based quality to these statements that should not be overlooked. While these statements reference past violent behavior, other participants in the virtual conversation have no way of verifying whether these incidents ever occurred. Nonetheless, each reference to past violence adds credibility and intensifies each statement. In some sense. the posts convey the following: “This is what we should do means this is what we might like to do, but it does not mean this is what we are going to do.” The examples of violent talk raise the question of whether we should treat these statements as literal declarations of a person’s intent to commit violence or rhetorical expressions meant to convey a particular identity. The latter position does not negate the influence of violent talk in terms of violent behavior, but rather, is cautious about assuming an overly deterministic relationship between violent words and violent action.^4^ Given that collective identity is diverse rather than uniform (Polletta and Jasper 2001) and determining who is a “real” white supremacist is not a clear-cut dichotomous assessment, violent talk may be one strategy white supremacists use to construct an authentic collective identity. From this perspective. the statements may not be indicative of a specific intent or plan to commit violence, but rather, provide important cultural clues regarding the centrality of violence in terms of identity construction and maintenance.

## 5. Discussion

The current study relied on ethnographic fieldwork with North American-based white supremacists to better understand the culture of violent talk. Overall, our investigation reveals that violent talk is pervasive across all types of white supremacist free spaces and functions as a rhetorical device that provides individuals with a sense of doing and an opportunity to express their frustrations and anger. While offensive and disturbing, these discussions do not necessarily involve a direct correspondence between a person’s words and future behavior. Rather, violent talk often involved “figures of speech” (Drew and Holt 1998), where individuals relied on fantasy-driven narratives and dramatic phrases to express emotions and achieve consistency between their personal and collective identities.

Based on our ethnographic evidence. the pervasiveness of violent talk is substantial, but what does this pervasiveness signal in terms of its importance? Some observers might contend that the ubiquity of violent talk suggests it is irrelevant and that all this talk amounts to is nothing more than hyperbole. Another quite different perspective offers that violent talk is important because it provides a clear indication of threat potential. Both perspectives are problematic and suffer from a lack of nuance. Violent talk is an important window into a person or group’s worldview (Blumer 1969) and can enhance the likelihood and success of implementation. Violent talk helps enculturate individuals through socialization processes by communicating values and norms. In turn, these values and norms are part of a process where in-group and out-group boundaries are established, potential targets for violence are identified and dehumanized, violent tactics are shared, and violent individuals and groups are designated as sacred (e.g., referring to individuals like Tim McVeigh, who committed the Oklahoma City bombing as a “martyr” or bestowing other violent perpetrators with titles such as “Saint” or “Sir”). In short, violent talk clearly plays an important role in terms of fomenting actual violence, but there is, nonetheless, an important distinction between talking and doing that can be overlooked when the words are as offensive as the ones we have described in this paper.

While talk provides actors with a cultural roadmap and observers with important cultural traces (Geertz 1973), talk cannot always be taken literally. For example, Goffman’s (1959) distinction between “frontstage” and “backstage” behavior suggests that an inconsistency between what people say and what people do is a defining feature of various social roles and institutional spheres. Since extremism, like all social roles, is a performance (Goffman 1959), successfully accomplishing the role of extremist may require the actor to engage in certain levels of violent talk. As such, talk becomes part of the performance as actors exchange extreme statements with an invisible “wink and nod,” signifying that the words are not always meant as literal expressions of future behavior. In this way, talk is strategic in an interactive sense but not necessarily a clear indication of plans for future violence. If a person defines violent talk as performance, then talk may provide consistency between thought and action often desired. In other words, if an actor views talk as a form of action, this may provide the necessary satisfaction to make further action unnecessary (i.e., talk is enough).

In addition to the performative nature of violent talk, these exchanges reveal important “therapeutic” markers useful for understanding the mindset and social landscape of the WSM and extremism more broadly. Paradoxically, in some cases, violent talk may decrease the likelihood of violence by providing extremists an opportunity to vent grievances. In some instances. the response to violent talk among white supremacists resembles a form of catharsis (Da Cunha and Orlikowski 2008).^5^ Although violent talk may not be therapeutic in the clinical sense, in certain situations (and for certain individuals), violent talk may generate responses such as laughter, which provide a sense of relief. As such, violent talk may act as a “stand-in” or substitute that allows white supremacists to express frustrations among themselves rather than through overt acts of violence against their perceived enemies. The moderating impact of violent talk, however, begs a related question: how long does violent talk as a cathartic release satisfy an extremist? There may exist a threshold in which people no longer feel satisfied and begin to redefine venting as “inaction” or not enough action. In this sense. the extremist may begin to feel that “talk is cheap” and it is time to “walk the walk” and, thus, align his/her behavior in a way that is consistent with violent talk.

While our findings emphasized similarities in violent talk across different social-spatial locations, future research should attend to differences that may be present in the nature of violent talk over time. This is especially relevant to the type of violent talk found on various social media platforms such as *4Chan* and *Telegram* and whether the violent talk on these platforms differs from earlier platforms like *White Revolution* and *Stormfront*. Much of the violent talk on these earlier platforms possessed a fantasy-driven quality, and, thus, it is important for additional studies to examine whether violent talk on newer digital platforms has retained this quality or assumed a more literal and practical quality.

The final point raises an interesting question: Are relatively higher levels of violent talk an indication of a lower level of threat as opposed to a higher one? There is precedent in the threat assessment literature that lends support to this possibility. For example, there is a common distinction between “hunters” and “howlers,” where the former includes individuals who intend to harm other people while the latter applies to individuals seeking attention (Calhoun and Weston 2008). In addition, a Secret Service study conducted by Borum et al. (1999) suggests individuals who make threats are not necessarily the individuals who pose the greatest risk and, in some cases, individuals who resort to violence do so without previously making direct threats. From this perspective, less talk may suggest the person is more comfortable with their role as an extremist and thus more inclined toward planned acts of violence. Interestingly, this hypothesis echoes the common folk wisdom that it is “always the quiet ones.”

The distinction between violent talk and action has important implications for homeland security and threat assessment professionals. Specifically, there is an important distinction between understanding the cultural significance of violent talk and using violent talk as a reliable indicator of threat. The idea of evaluating and predicting threats is an age-old consideration. From an evolutionary perspective, human survival demands the assessment of threat (Flannelly et al. 2007). In an age where terrorism generates the level of concern that it does today, threat assessment considerations have taken on an even greater prominence (Clemmow et al. 2020; Logan and Lloyd 2019). In the United States, for example, an entire industry of threat assessment offers advice to employers, schools, and a variety of other social institutions about detecting potential perpetrators (Mueller 2010). In response to 9/11 and concerns over homegrown violent jihadis. the Federal Bureau of Investigation and other law enforcement agencies have aggressively monitored and searched for indicators of threat among “splinter cells” and “lone wolves.” Violent talk observed or reported either in person or online is used, in some cases, as “red flags” and receives additional scrutiny from law enforcement authorities. When violent talk is perceived to constitute an indication of an ongoing threat to commit violence, law enforcement may approach the person(s) and attempt to entice further radicalization by offering explosives or weapons and suggesting plots to commit violence.

In some cases. the “agent provocateurs” (Marx 1974) walk a fine line between legal investigation and entrapment. What is more clear, however, is that agent provocateurs have wide latitude in performing their role and use this discretion to interpret violent talk in ways that confirm investigative assumptions regarding potential threats as well as engage in radical behavior designed to accelerate the threat of suspects who are under investigation (Aaronson 2013; McCulloch and Wilson 2016). When talk is used as a threat indicator, however. the spontaneity of talk is often glossed over. The dynamic emergence of violent talk challenges the logic of prediction which underscores its limit as a reliable marker of threat. At the same time, while violent talk may erode civic discourse, repressive responses to violent talk threaten civil liberties and undermine American constitutional protections.

## Footnotes

1 We use talk and conversation interchangeably and define them as involving face-to-face informal interactions where two or more individuals exchange words with each other for the purpose of communication (Heritage 1984).

2 The states are Alabama, Arizona, California, Georgia, Idaho, Illinois, Kansas, Maine, Michigan, Montana, New Jersey, New York, Ohio, Oregon, Pennsylvania, Texas, and Washington.

3 In the case of widely known white supremacist groups. the names of the organizations and their representatives are left unchanged. In other cases, pseudonyms are used.

4 One could certainly argue that violent words especially ones expressed over the Internet are an example symbolic violence (see Bourdieu 1991).

5 See Scheff (2007) for an in-depth discussion of the current status of catharsis as a concept.
